# A reliable method for intracranial electrode implantation and chronic electrical stimulation in the mouse brain

**DOI:** 10.1186/1471-2202-14-82

**Published:** 2013-08-06

**Authors:** Melanie Jeffrey, Min Lang, Jonathan Gane, Chiping Wu, W McIntyre Burnham, Liang Zhang

**Affiliations:** 1 Toronto Western Research Institute, University Health Network, Toronto, Ontario, Canada; 2 Department of Pharmacology, Toronto, Ontario, Canada; 3 Department of Medicine (Division of Neurology), University of Toronto, Toronto, Ontario, Canada; 4 University of Toronto Epilepsy Research Program, Toronto, Ontario, Canada

**Keywords:** Glue, Kindling, Mice

## Abstract

**Background:**

Electrical stimulation of brain structures has been widely used in rodent models for kindling or modeling deep brain stimulation used clinically. This requires surgical implantation of intracranial electrodes and subsequent chronic stimulation in individual animals for several weeks. Anchoring screws and dental acrylic have long been used to secure implanted intracranial electrodes in rats. However, such an approach is limited when carried out in mouse models as the thin mouse skull may not be strong enough to accommodate the anchoring screws. We describe here a screw-free, glue-based method for implanting bipolar stimulating electrodes in the mouse brain and validate this method in a mouse model of hippocampal electrical kindling.

**Methods:**

Male C57 black mice (initial ages of 6–8 months) were used in the present experiments. Bipolar electrodes were implanted bilaterally in the hippocampal CA3 area for electrical stimulation and electroencephalographic recordings. The electrodes were secured onto the skull via glue and dental acrylic but without anchoring screws. A daily stimulation protocol was used to induce electrographic discharges and motor seizures. The locations of implanted electrodes were verified by hippocampal electrographic activities and later histological assessments.

**Results:**

Using the glue-based implantation method, we implanted bilateral bipolar electrodes in 25 mice. Electrographic discharges and motor seizures were successfully induced via hippocampal electrical kindling. Importantly, no animal encountered infection in the implanted area or a loss of implanted electrodes after 4–6 months of repetitive stimulation/recording.

**Conclusion:**

We suggest that the glue-based, screw-free method is reliable for chronic brain stimulation and high-quality electroencephalographic recordings in mice. The technical aspects described this study may help future studies in mouse models.

## Background

Electrical kindling of limbic structures in rodents is a widely used model for complex partial seizures with secondary generalization [1-4]. This model requires implantation of intracranial electrodes and subsequent brain stimulation for several weeks. The majority of previous studies have employed this model in rats, using anchoring screws and dental acrylic to secure the implanted electrodes [5-9]. Recent studies have increasingly applied the kindling model in mice largely due to the availability of transgenic mouse strains [10-13].

In general, it is a technical challenge to firmly mount intracranial electrodes in mice with the anchoring screw/dental acrylic approach, since the mouse’s thin skull may not be strong enough to accommodate the anchoring screws. As an alternative approach, we have previously developed a screw-free, glue-based method for implanting intracranial electrodes in mice [14]. Using this method, we have carried out chronic intracranial electroencephalographic (EEG) recordings in several mouse models [15-20]. In these studies, a pre-assembled electrode array was used. While the glue-based method can be used for implanting bipolar stimulating electrodes in the mouse brain [21], its validity for the chronic brain stimulation in mice that is required for kindling has not been vigorously assessed. The aim of our present study is to address this issue. We thus further modified the glue-based implantation method and conducted hippocampal electrical kindling in adult mice. EEG discharges and behavioral seizures were consistently observed in kindled mice. Importantly, no animal encountered infection in the implanted area or a loss of implanted electrodes after 4–6 months of repetitive stimulation/recording. We thus suggest that the glue-based method is reliable for chronic brain stimulation and high-quality EEG recording in mouse models.

## Results

### Long-term stability of implanted electrodes and animal behavioral outcomes

**Figure 1:**
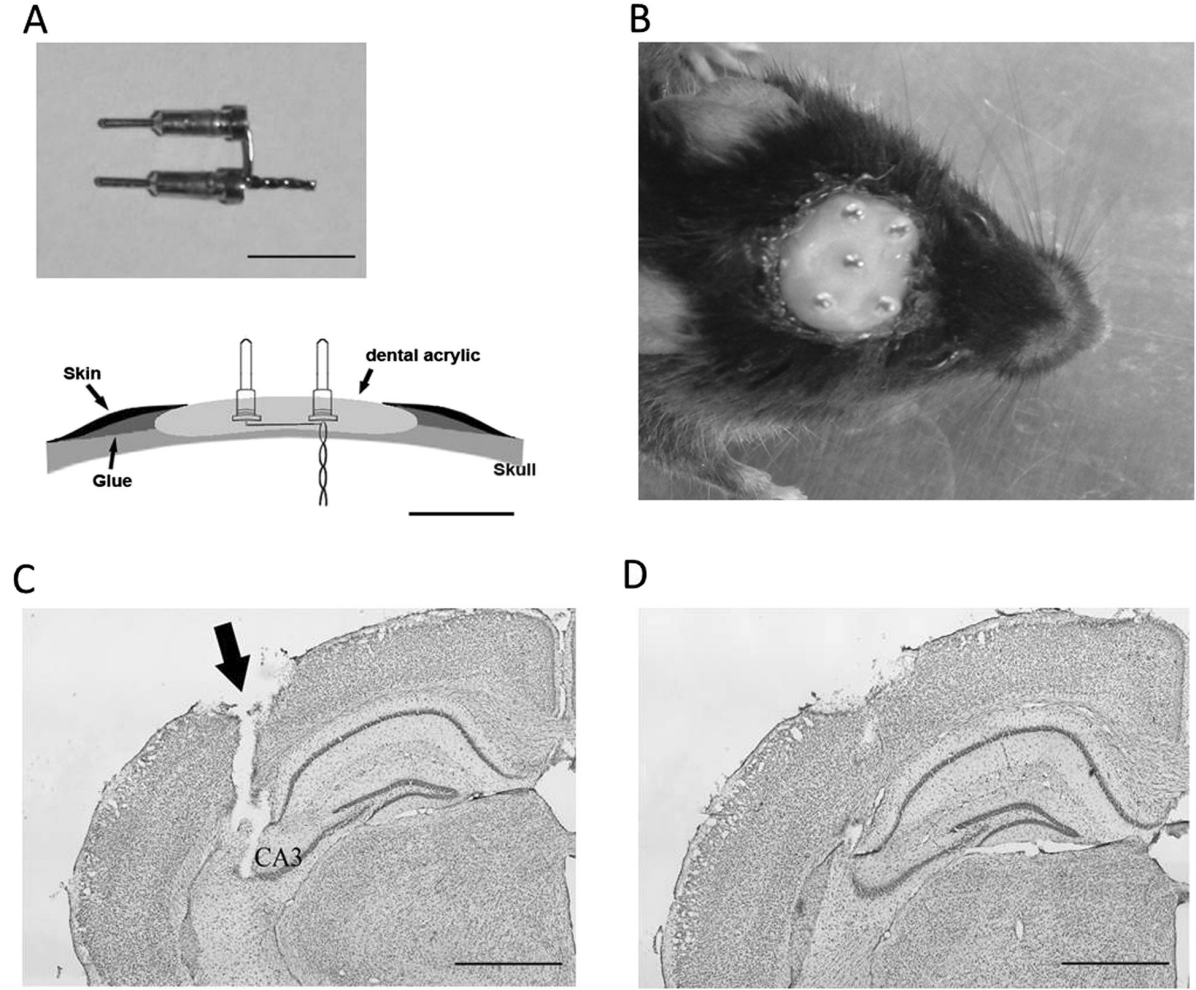
**Illustration of connecting pins and mounted electrodes. A,** a schematic illustration of electrode mounting and an image of connecting pins and a bipolar electrode. **B,** a photograph of a subject implanted. **C-D,** images of adjacent brain sections collected from a mouse. The animal was euthanized 3 months after electrode implantation. Note the track of an implanted CA3 electrode **(**arrowed, **C)** and densely packed hippocampal neurons in the area near the implanted electrode and in the adjacent section **(D)**. Calibrations: 5 mm in **A** and 1 mm in **C**-**D**.

25 adult male mice (initial ages of 6–8 month-old) underwent implantation of bipolar electrodes in bilateral hippocampal CA3 (Cornu Ammonis area 3). These electrodes were secured onto the skull with a modified version of the screw-free and glue-based method [14,21]. Electrode construction, assembly and mounting are schematically illustrated in Figure 1A. The surgical procedure for electrode implantation is detailed in the Methods. Of the 25 animals implanted, we observed no evident behavioral abnormalities or spontaneous seizures in the absence of kindling stimulations. Importantly, no animal encountered local infection in the implanted area or a loss of implanted electrodes after 4–6 months of repetitive stimulation and recordings.

**Figure 2:**
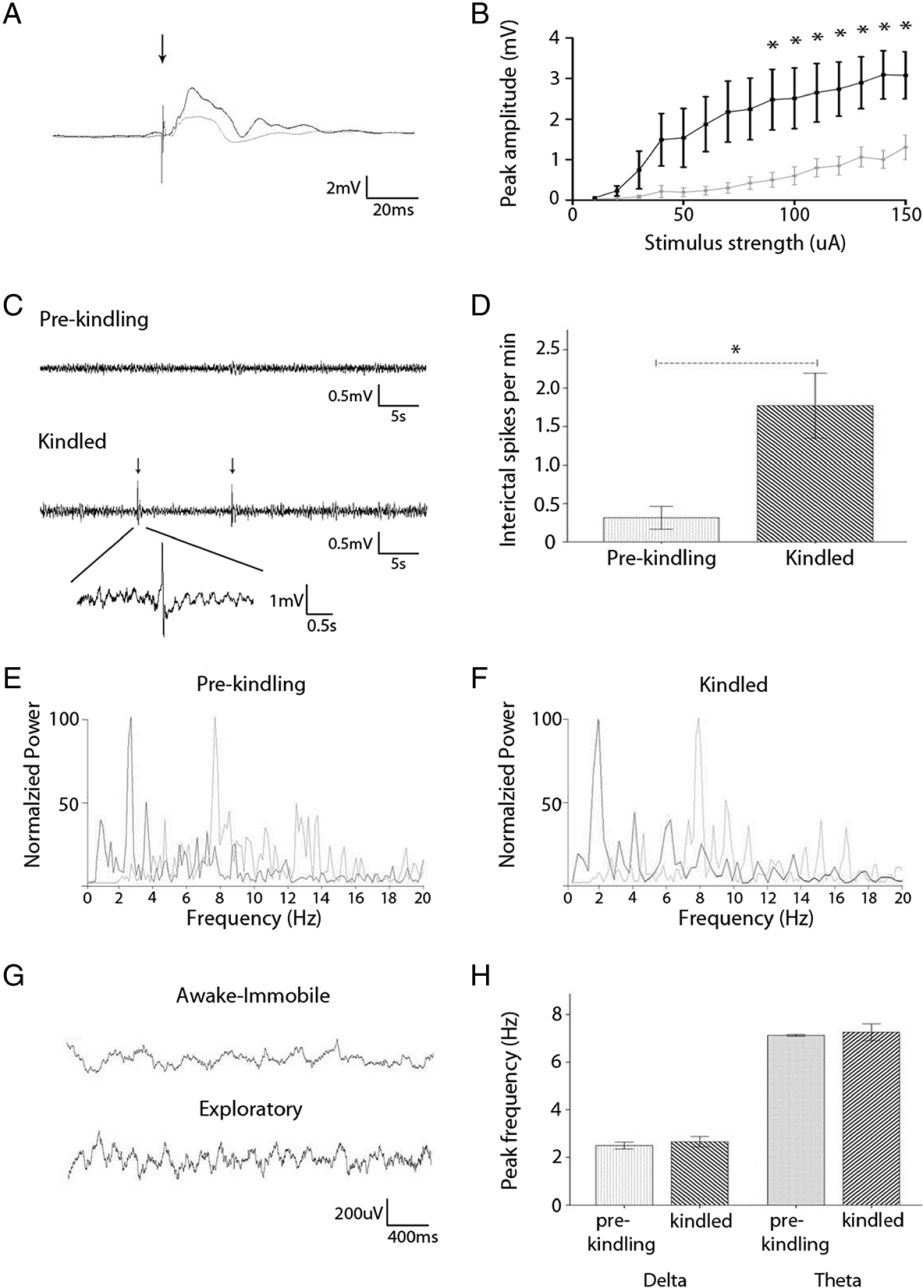
**Electrophysiological verifications of implanted electrodes. A,** representative field potentials collected from a mouse at the beginning the kindling procedure (gray) and after reaching a fully kindled state (black). These field potentials were evoked by unilateral hippocampal CA3 stimulation (arrowed) and recorded from the contralateral CA3 area. Each illustrated trace was averaged from 5 consecutive responses. The artifact of the stimulation is indicated (arrow). **B,** the amplitudes of CA3 field potentials measured from 5 animals pre-kindling (gray) and after kindled (black). *, initial vs. kindled responses; p < 0.05, paired t test. **C,** interictal-like hippocampal spikes (arrowed) recorded from a kindled animal. **D,** incidences of hippocampal spikes measured pre-kindling and after fully kindled. *, p = 0.013, paired t-test, n = 7. **E**-**F,** representative spectral plots were generated from EEG signals that were recorded from an animal **(G)** pre-kindling **(E)** and after fully kindled **(F)**. The spectral plots were generated from EEG segments of 30-second or 5-second during wake immobile (black) or exploratory (gray) behavior. Power was normalized to the peak of the delta band (0.5-4 Hz). **G,** representative EEG traces collected from the same animal after fully kindled. **H,** peak frequencies of hippocampal theta rhythms (5–12 Hz) and delta irregular activities (0.5-4 Hz) were measured from 7 animals. The peak frequencies of hippocampal theta and delta activities were not significantly altered in kindled animal relative to pre-kindling measurements (p = 0.723 and p = 0.524, paired test).

The locations of implanted electrodes were initially verified by *in vivo* electrographic activities and later, histological assessments. In response to a single stimulation of the unilateral CA3 area, synaptic field potentials were reliably recorded from the contralateral CA3 area (Figure 2A). The amplitudes of these field potentials increased with strong stimuli and reached a near plateau level at the stimulation intensity of ≥100 μA (Figure 2B). No animals showed epileptic responses (such as long-lasting multi-spike waveforms) following a single stimulation. In addition, the animals exhibited hippocampal “physiological” activities similar to those we and others have previously observed in mice [15-20,22]. Specifically, CA3 recordings revealed rhythmic activities in the theta band (5–12 Hz) while the animals moved/explored local environments and irregular activities in the delta band (0.5-4 Hz) during immobility or sleep (Figure 2E-H). The peak frequencies of hippocampal theta and delta activities were not significantly changed in kindled animals (n = 7 mice; Figure 2H). As bilateral hippocampi are strongly interconnected via ventral and dorsal hippocampal fissures and the CA3 area is critical for the generation of hippocampal EEG rhythms and epileptiform activities [23], the above data provide electrographic evidence for accurately implanted CA3 electrodes.

The locations of implanted CA3 electrodes were histologically confirmed in 6 animals. The tracks of implanted electrodes were recognizable in brain sections that corresponded to the desired stereotaxic coordinates (Figure 1C). There was no evident loss of hippocampal neurons in the areas near the implanted electrodes or in adjacent brain sections (Figure 1D). Taken together these observations with the electrographic data above, we suggest that the screw-free and glue-based method is suitable for reliable implantations of bipolar stimulating/recording electrodes in adult mice.

### Seizure activities induced by hippocampal kindling

We used a standard kindling protocol in the present experiments [24,25]. The animals were considered fully kindled if they exhibited stage 5 behavioral seizures on 3 consecutive days. Of 25 animals tested, 21 animals were fully kindled between 6 and 26 stimulations (mean ± SD 12.14 ± 6.41). Two of the remaining 4 animals failed to reach a fully kindled state due to contamination of implanted electrodes (see Discussion) and the other two died spontaneously with underlying causes presently unknown.

**Figure 3:**
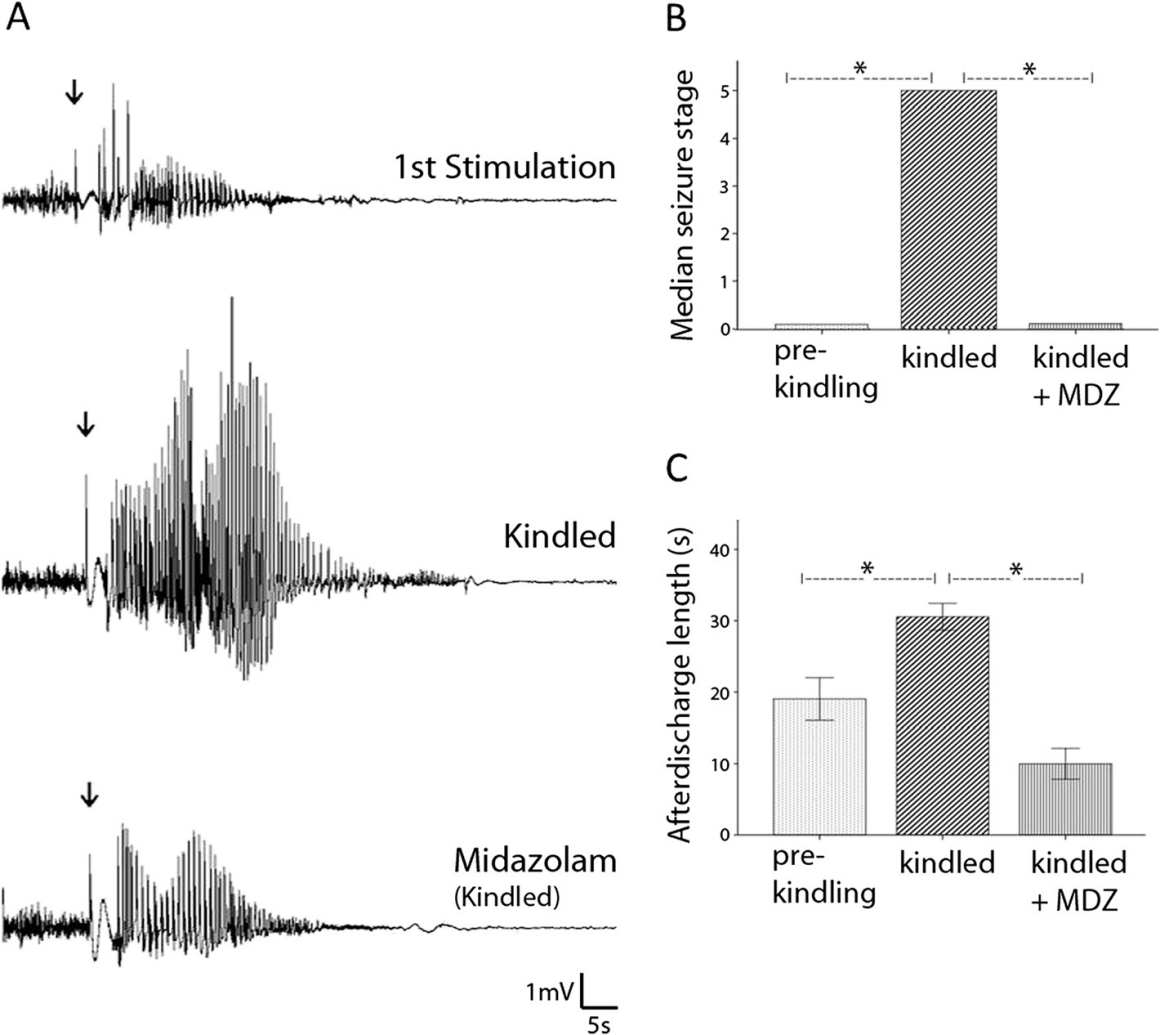
**EEG discharges and motor seizures induced by hippocampal kindling. A,** afterdischarges recorded from a mouse after the first stimulation (top) and fully kindled following an intra-peritoneal injection of saline (middle) or midazolam (MDZ; 2 mg/kg, bottom). The afterdischarges were evoked by unilateral CA3 stimulation (60 Hz for 2 seconds, indicated arrows) and recorded from the contralateral CA3 area. **B,** seizure stages assessed from 6 animals. *, p = 0.008 or p = 0.014; Wilcoxon signed rank test. **C,** afterdischarges measured from same 6 animals. *, p = 0.014; paired t tests.

In addition to consistent expression of the stage 5 behavioral seizures, the kindled animals showed significant changes in hippocampal electrographic activities. First, the evoked field potentials were greatly enhanced in the kindled animals as compared to their own baselines (Figure 2A). Such enhancements were recognizable at all stimulation intensities examined (Figure 2B), suggesting an overall increase of hippocampal synaptic strength induced by the kindling process. Secondly, the afterdischarges (ADs) were greatly increased in amplitude and duration in the kindled animals (Figure 3A, C). Finally, interictal-like hippocampal spikes, similar to those previously described [26-29], were frequently observed in the kindled animals but were either absent or barely detectable in the same animals during baseline monitoring (Figure 2C-D).

The behavioral seizures and ADs observed from the kindled animals were sensitive to inhibition by midazolam, a fast acting benzodiazepine analogue recommended for the treatment of status epilepticus in clinical practice [30,31]. Midazolam was administered via intra-peritoneal injections at a dose of 2 mg/kg, 15 minutes prior to the hippocampal stimulation. In the 6 kindled animals tested, ADs and behavioral seizures were significantly attenuated following midazolam injections compared to those observed following the saline injections (Figure 3A-C). Together, the above observations suggest that a robust epileptic process is established in the kindled mice.

## Discussion

Using a modified version of the screw-free and glue-based method, we implanted bipolar electrodes in bilateral hippocampal CA3 areas. In our experiments, no animal had adverse health-related events, spontaneous seizures, or abnormal behavior in the absence of electrical stimulation. There was no local infection in the implanted area and no loss of implanted electrodes after repetitive stimulations and recordings for 4–6 months. The kindled animals exhibited consistent stage 5 behavioral seizures, robust ADs (Figure 3A-D), and frequent interictal-like spikes (Figure 2C-D). The AD and behavioral seizures were sensitive to suppression by midazolam (Figure 3A-C). We thus suggest that the modified glue method is reliable for intracranial electrode implantation, chronic brain stimulation and high-quality EEG recordings in mice. Specific technical issues of the modified method are discussed below.

In our previous studies [14-20], we used a pre-constructed electrode array with a plastic base and applied glue between the plastic base and skull surface. Because the glue bonds the plastic base strongly to the skull, the electrode array can be firmly mounted onto the skull without using anchoring screws. This method is convenient for implanting the electrode array with 2–3 monopolar electrodes, and allows stable intracranial EEG recordings in mouse models [15-20]. This method, however, is limited for implanting bipolar electrodes that are aimed to stimulate and/or record from spatially separated brain structures, since these electrodes are difficult to assemble in a compact array. In addition, due to the curvature of the skull, these electrodes need to be individually inserted into the brain for accurate positioning.

We thus used a modified approach in the present experiments. We first glued a plastic base onto the skull surface and drilled small holes through the plastic base and underneath skull. We then individually positioned bipolar electrodes in the brain and overlaid dental acrylic onto the plastic base covering the implanted electrodes. When hardened, the dental acrylic and the plastic base beneath the acrylic were strongly bonded; this secured the implanted electrodes (Figure 1A-B). This modified approach is successful because dental acrylic denatures the plastic base before hardening and then bonds to the plastic base after hardened. The implanted electrodes are secured onto the skull when the dental acrylic has hardened. Experience is needed, however, to promptly overlay the dental acrylic onto the plastic base, and to carefully cover the implanted electrode bases, before the acrylic hardens. We used a dental acrylic with hardening time of 6–9 minutes in the present experiments to have sufficient time to apply the dental acrylic.

In the present experiments, the pins used to connect electrodes were detached from standard IC sockets (Figure 1A). We chose these pins because the IC sockets are widely available from commercial sources at a low cost. In addition, these pins are relatively strong and offer good contacts when connected. Thus, although animals frequently push or press these pins against their cages or metal food racks, the implanted electrodes maintain integrity for chronic stimulation and/or recordings. Care should be taken, however, when detaching these pins from wire sockets, to preserve a gasket-like piece in the female end of individual pins.

We implanted bipolar electrodes in bilateral hippocampal CA3 areas in the present experiments. This experimental design was used to test the possibility of stimulating two brain regions, and/or to increase the probability of electrical kindling in individual animals in the event that one bipolar electrode fails. The latter case was encountered in our initial experiments, likely due to electrode contamination that occurred during and/or after implantation. Such a problem did not occur in our later experiments, by visual inspection of electrode tips under a microscope and measurements of electrode conductivity before implantation. Therefore, a careful examination of the constructed electrodes prior to surgical implantation may greatly increase the success rate of chronic brain stimulation or recording.

Several previous studies have used cyanoacrylate-type glue alone or together with dental acrylic to secure EEG recording as well as stimulating electrodes in mouse models [32-34]. However, the underlying procedures were not detailed. It is possible that the electrode implantation methods used in these previous studies and our present experiments may share some common features. By detailing the glue-based implantation method, our present study may support the previous studies and provide complementary technical aspects for further employment of this approach in mouse models.

## Conclusion

We suggest that the glue-based and screw-free implantation method is reliable for chronic brain stimulation and high-quality EEG recordings in mice. The method may also be used, with some modifications, to implant other probes or infusion tubes in the mouse brain.

## Methods

### Animals

Male C57 black mice (initial ages of 6–8 months, Charles River Laboratory, Quebec, Canada) were used in the present experiments. These animals were housed in a vivarium that was maintained at 22°C with 12-hour light and dark cycles. Food and water were available *ad libitum*. Stimulations and recording experiments were done between 12-5 pm. All experimental procedures described below have been reviewed and approved by the Animal Care Committee of the University Health Network in accordance with the guidelines of the Canadian Council on Animal Care.

### Electrode construction

All electrodes were made of polyamide-insulated stainless steel wires (outer diameter 200 μm, Plastics One, Ranoake, VA). Twisted bipolar wires were used for stimulation and recording, with their intracranial tips ~100 μm apart. The extracranial tips of the twisted wire assembly were soldered to the female ends of two connecting pins, with one wire bent into an L-shape to separate the connecting pins (Figure 1A). Care was taken to fully remove the insulation layer before soldering. A liquid solder (Soldering Liquid Flux, Certanium Alloys and Research Company, Cleveland OH, USA) or weak phosphoric acid was used to ensure good contact between the stainless steel wire and the connecting pin. A single wire was similarly soldered to a single pin for reference. The resistance of each constructed wire electrode was ≤1.0 Ω. After being soldered to the connecting pins, the bipolar wires were then cut to ~3 mm in length to target the desired hippocampal CA3 area (see below). The single monopolar wire was cut to ≤0.5 mm for epidural position of the reference electrode. These electrodes were cleaned with 75% alcohol and stored in a sterilized glass vial until use. The connecting pins were detached from standard IC sockets (Samtec series SS socket strips, SS-132-G2, Electrosonic, Toronto, Ontario, Canada). These pins are 7.5 mm long in total; the male end of the pin is 3.2 mm long. The outer diameter is 1.8 mm or 0.5 mm for female or male end of the pin respectively (Figure 1A). Measured after being dissected out from implanted animals (n = 3), the total weight of implanted electrode assembly, including electrode wires, plastic base and dental acrylic (see below), was 0.50-0.52 grams. As adult mice were used in the present experiments, the weight of implanted electrode assembly was ≤2% of animal body weight.

### Surgery for electrode implantation

Surgical procedures were modified from those we previously described [14,21]. Briefly, the animal was anaesthetized with 2% isoflurane and then placed on a stereotaxic frame. After skin incision and exposure of the skull surface, the tip of a mini drill bit (see below) was aimed to bregma via a micromanipulator. After determining the bregma position, the drill bit was moved up but its X-Y position was unchanged, and a thin plastic base then was glued onto the skull surface. After the glue had cured, three small holes were then drilled through the plastic base and the skull according to the stereotaxic coordinates of the hippocampal CA3 area (bregma −2.5 mm, lateral ±1.3 mm, and a depth of 3.2 mm [35]). The reference electrode was positioned at bregma 1 mm, lateral 2.0 mm and a depth of 0.7 mm. The electrode depths were adjusted to accommodate for the thickness (200 μm) of the plastic base.

The plastic base was cut from a curved part of plastic weighting boats (polystyrene antistatic weighting dishes, Fisher Scientific, cat#08-732-115). The weighting boats were 140x140 mm in length-width and 25 mm in depth, with thickness of ~200 μm. The plastic base was soft and could be gently pressed to accommodate curvature of the skull, and thus bound tightly with the skull surface after being glued. We used a cyanoacrylate-type glue (Insta-cure+, cure time 5–15 seconds, made in U.S.A., cat# BSI-106C; obtained from Canadian Hobbycraft, Concord, Ontario, Canada). A motorized drill (model FM3545, Foredom Electric, Bethel, CT, USA) and a mini drill bit (part 115603, Ball Mills Carbide, CircuitMedic, Haverhill, MA, USA) were used to drill small holes (≤0.5 mm) through the skull. These holes were large enough for inserting the electrodes, but small enough to prevent dental acrylic leakage into the brain (see below).

Micromanipulators were also used to individually insert the bipolar electrodes into bilateral hippocampal CA3 areas. After positioning and holding these electrodes with the micromanipulators, dental acrylic was overlaid onto the plastic base such that the bases of the connecting pins were covered by the dental acrylic (Figure 1B). Care should be taken to apply acrylic so as not to interfere with electrode positions and to contaminate connecting pins. We used a dental acrylic with hardening time of 6–9 minutes (Jet Tooth Shade, Reference No. 1404; Lang Dental Mfg. Co., Inc., Wheeling, IL, USA) to carefully cement the implanted electrodes. After the dental acrylic had hardened, the electrodes were released from the micromanipulators. The incised skin was then glued to the dental acrylic (Figure 1A,1B), which prevents infection in the implanted area. The animals were released from the stereotaxic frame and allowed to recover for at least one week before further experimentation.

### Electrical stimulation

Unilateral CA3 stimulation was conducted in all present experiments. Constant square-wave current pulses (duration of 0.5 ms, intensities of 10–150 μA) were generated from a Grass stimulator and delivered through an isolation unit (model S88H, Grass Medical Instruments, Warwick RI, USA). To establish an input–output plot for evoked CA3 field potentials, a single stimulation was applied every 30 seconds at intensities of 10–150 μA (10 μA increments; 5 consecutive responses at a given intensity). A standard kindling protocol [24,25] was used. Animals were considered fully kindled after consecutive stage 5 seizures on 3 consecutive days.

### Recordings and measurements

All recordings were from the CA3 area contralateral to the CA3 stimulation site. Signals were recorded via a 2-channel microelectrode AC amplifier (model 1800, A-M Systems, Carlsborg, WA, USA), with the input frequency band set in the range of 0.1-1000 Hz, and the amplification gain at 1000×. The signals were digitized at 5000 Hz (Digidata 1440A, Axon Instruments/Molecular Devices, Union City, CA, USA). Pclamp software (Version 9 or 10; Axon Instruments/Molecular Devices) was used for data acquisition, storage and analyses.

The amplitudes of evoked field potentials were measured from averaged traces (5 consecutive responses) at a given stimulation intensity. To measure hippocampal rhythmic activities associated with “active” and “inactive” behaviors, stable data segments of 5–10 seconds or 30–60 seconds were selected while the animals were moving/exploring or immobile/asleep. Spectral plots were generated from these data segments and peak frequencies were measured from the spectral plots for individual animals. To detect interictal-like spikes, individual animals were recorded for up to four hours before kindling was initiated. After animals were fully kindled, interictal spikes were recorded for 4–6 hours after the most recent ADs. A spike was only counted if its amplitude was large (≥2 times the amplitude of the background signal) and its waveform was similar to those previously described [26-29]. To minimize interference of movement-related artifacts, the rates of interictal spikes were measured in the periods (1–2 hours) while the animals were immobile/asleep. To measure AD durations, corresponding EEG data were treated with a 0.5 Hz high-pass (Bessel) filter to reduce slow drifts in EEG signals.

### Behavioral assessments

Animal’s behaviors were recorded with a high definition camera and analyzed by blinded experimenters. Behavioral seizures were scored using a modification of the Racine scale for the mouse [25]: stage 0, no response or behavioral arrest; stage 1, chewing or head-nodding; stage 2, chewing and head nodding; stage 3, single or bilateral forelimb clonus; stage 4, bilateral forelimb clonus and rearing; stage 5, loss of righting reflex (falling).

### Brain histology

Histological experiments were conducted as we previously described [15,17]. Briefly, the animals were anesthetized with pentobarbital (70 mg/kg, i.p.) and trans-cardiacally infused with saline and then with 10% phosphate-buffered formalin solution before decapitation. Cryostat coronal sections 30 μm thick were obtained throughout the brain, stained with cresyl violet, and examined under a light microscope.

### Statistical analysis

Statistical tests were performed with SPSS software (Version 20, SPSS Statistics, IBM). Data are presented as mean and standard error of mean (M ± SE) throughout the text and figures except where indicated.

## Abbreviations

AD: After-discharges; CA3: Cornu ammonis 3 area; EEG: Electroencephalography.

## Competing interests

The authors of this study declare that they have no competing interests.

## Authors’ contributions

MJ and ML made equal contributions to this study. All authors were involved in experimental design, background/literature search and conceptualizations of this study. Specifically, MJ, ML and JG conducted experiments and data analysis. CW constructed electrodes and conducted surgeries for electrode implantation. MJ, ML and LZ assembled manuscript. MB edited the manuscript. All authors have read and approved the final manuscript.

## Authors’ information

MJ-PhD student at the Department of Pharmacology, University of Toronto.

ML-MSc, Research fellow, Toronto Western Research Institute.

JG-Undergraduate student at the University of Toronto.

CW-Research associate at the Toronto Western Research Institute.

MB-Professor at the Department of Pharmacology, University of Toronto.

LZ-Research Scientist at the Toronto Western Research Institute.
